# Alternative metrics for characterizing longer-term clinical outcomes in difficult-to-treat depression: I. Association with change in quality of life

**DOI:** 10.1017/S0033291722003798

**Published:** 2023-10

**Authors:** Harold A. Sackeim, A. John Rush, Teresa Greco, Mei Jiang, Sarah Badejo, Mark T. Bunker, Scott T. Aaronson, Charles R. Conway, Koen Demyttenaere, Allan H. Young, R. Hamish McAllister-Williams

**Affiliations:** 1 Departments of Psychiatry and Radiology, Columbia University, New York, NY, USA; 2 Duke-NUS Medical School, Singapore; 3 Department of Psychiatry and Behavioral Sciences, Duke University, Durham, NC, USA; 4 LivaNova PLC, Milan, Italy; 5 Jazz Pharmaceuticals PLC, Milan, Italy; 6 LivaNova USA PLC, Minneapolis, MN, USA; 7 LivaNova USA PLC, Houston, TX, USA; 8 Department of Clinical Research, Sheppard Pratt Health System, Baltimore, MD, USA; 9 Department of Psychiatry, Washington University in St. Louis, St. Louis, MO, USA; 10 Faculty of Medicine KU Leuven, University Psychiatric Center KU Leuven, Leuven, Belgium; 11 Department of Psychological Medicine, Institute of Psychiatry, Psychology and Neuroscience, King's College London, London, UK; 12 South London and Maudsley NHS Foundation Trust, Bethlem Royal Hospital, Beckenham, UK; 13 Northern Centre for Mood Disorders, Translational and Clinical Research Institute, Newcastle University, UK, and Cumbria, Northumberland, Tyne and Wear NHS Foundation Trust, Newcastle upon Tyne, UK

**Keywords:** difficult-to-treat depression, effect size, efficacy, intervention research, metrics, outcome measures, quality of life, remission, treatment-resistant depression

## Abstract

**Background:**

In difficult-to-treat depression (DTD) the outcome metrics historically used to evaluate treatment effectiveness may be suboptimal. Metrics based on remission status and on single end-point (SEP) assessment may be problematic given infrequent symptom remission, temporal instability, and poor durability of benefit in DTD.

**Methods:**

Self-report and clinician assessment of depression symptom severity were regularly obtained over a 2-year period in a chronic and highly treatment-resistant registry sample (*N* = 406) receiving treatment as usual, with or without vagus nerve stimulation. Twenty alternative metrics for characterizing symptomatic improvement were evaluated, contrasting SEP metrics with integrative (INT) metrics that aggregated information over time. Metrics were compared in effect size and discriminating power when contrasting groups that did (*N* = 153) and did not (*N* = 253) achieve a threshold level of improvement in end-point quality-of-life (QoL) scores, and in their association with continuous QoL scores.

**Results:**

Metrics based on remission status had smaller effect size and poorer discrimination of the binary QoL outcome and weaker associations with the continuous end-point QoL scores than metrics based on partial response or response. The metrics with the strongest performance characteristics were the SEP measure of percentage change in symptom severity and the INT metric quantifying the proportion of the observation period in partial response or better. Both metrics contributed independent variance when predicting end-point QoL scores.

**Conclusions:**

Revision is needed in the metrics used to quantify symptomatic change in DTD with consideration of INT time-based measures as primary or secondary outcomes. Metrics based on remission status may not be useful.

## Introduction

A substantial proportion of patients in a major depressive episode (MDE) do not remit despite multiple, well-delivered acute antidepressant treatments (Jaffe, Rive, & Denee, 2019; Rush et al., 2006c). For other patients, lack of remission may be linked to factors that impede the delivery of ‘adequate’ antidepressant trials, including pervasive intolerance or non-adherence (Chekroud et al., 2018; Corey-Lisle, Nash, Stang, & Swindle, 2004; Murphy, Kremer, Rodrigues, & Schatzberg, 2003), or factors such as cost and accessibility (Gauthier et al., 2017; Lamb, Bower, Rogers, Dowrick, & Gask, 2012). Still other patients remit with acute antidepressant treatments, but sustained benefit is not achieved due to rapid and/or frequent relapse (Aaronson et al., 2021; Rush et al., 2006c; Sackeim et al., 2007; Singh, Fedgchin, Daly, & Drevets, 2020). The concept of treatment-resistant depression (TRD) focuses on those who do not benefit from adequate acute antidepressant treatments (Sackeim, 2001; Thase & Rush, 1995). The recently introduced heuristic of difficult-to-treat depression (DTD) is conceptually broader and applies to individuals who do not achieve or sustain remission regardless of cause (McAllister-Williams et al., 2020; Rush et al., 2022; Rush, Thase, & Dube, 2003a).

The parsing of the treatment of depressive illness into distinct acute, continuation, and maintenance phases and the traditional outcome metrics used to document treatment efficacy are derived from the study of treatment-responsive populations and may not be applicable in DTD (Frank et al., 1991; Rush et al., 2006b). For example, the efficacy of acute phase antidepressant treatment is evaluated by determining the extent of symptom reduction at a pre-specified endpoint, e.g. after 4–12 weeks of an acute pharmacological, psychotherapeutic, or neuromodulatory intervention (Klein, Gittelman, Quitkin, & Rifkin, 1980; Prien, Carpenter, & Kupfer, 1991). The magnitude of symptom reduction and binary classifications of response and remission are based on comparison of this single end-point (SEP) ‘snapshot’ of symptom severity following acute phase treatment to a pre-treatment, baseline assessment. Durability of benefit is determined by identifying instances of relapse/recurrence during continuation or maintenance therapy of individuals who responded or remitted during the acute phase (Prien et al., 1984; Prien & Kupfer, 1986). The binary classifications of relapse and recurrence are also based on an SEP comparison of the change in symptom severity since the end of acute phase treatment.

This methodology is problematic in DTD for multiple reasons. First, the binary classifications that designate successful acute clinical outcome, response and remission, may not be useful since they apply infrequently and often only transiently. By definition, patients with DTD have not achieved sustained remission of depressive symptoms (McAllister-Williams et al., 2020). Especially in the context of failures of multiple adequate antidepressant treatments, such patients have reduced likelihood of achieving response, let alone remission, with novel interventions (Kraus, Kadriu, Lanzenberger, Zarate, & Kasper, 2019; Rush et al., 2006c; Sackeim et al., 2019). Nonetheless, in patients with DTD a more modest but sustained reduction in symptom severity may result in improved quality of life (QoL) and become the primary goal of treatment (Conway et al., 2018). In other words, in DTD patients the classifications of response and remission at a single point in time may be insufficiently sensitive in identifying those who achieve meaningful QoL improvement because of an intervention.

The assessment of durability of benefit is also problematic. Traditionally, durability of benefit has been defined by the relapse/recurrence rate during continuation/maintenance treatment in patients who met response or remission criteria following acute phase treatment (Frank et al., 1991; Paykel, 2001; Prien et al., 1984; Prien & Kupfer, 1986; Rush et al., 2006b). Most patients with DTD are excluded from long-term follow-up studies precisely because they do not achieve the traditional threshold of symptomatic improvement required to be monitored for relapse. Nonetheless, durability of benefit is of particular concern in their management. Since the depressive symptoms are often chronic, the durability of improvement is a fundamental consideration, particularly when interventions yield only modest acute gains, are expensive or resource intensive, or when a long period of treatment is required to achieve the benefit (Faxon et al., 2004; Kumar et al., 2019; Shiroma et al., 2020).

Another set of concerns apply to the use of SEP assessment to capture the impact of interventions on depressive symptoms. Traditionally, continuous or categorical measures of acute outcome reflect symptom severity at a fixed time point, independent of or in relation to a pre-treatment baseline. Similarly, relapse and recurrence also rely on SEP assessment of symptom severity relative to a post-acute treatment baseline. This SEP methodology presumes that a ‘snapshot’ is a reliable and stable indicator of clinical state. This assumption may not hold in DTD, where subgroups may have increased temporal variability in clinical state due to spontaneous fluctuation (e.g. circadian effects, frequent relapse) or as a result of concomitant treatments, co-morbidities, side effect burden, and environmental stressors (Bowen, Wang, Balbuena, Houmphan, & Baetz, 2013; Broome, Saunders, Harrison, & Marwaha, 2015; Witte, Fitzpatrick, Warren, Schatschneider, & Schmidt, 2006). It has also been long contended that improvement in QoL lags behind symptom change and that sustained symptomatic improvement is critical (Hofmann, Curtiss, Carpenter, & Kind, 2017; Megari, 2013; Paykel, 2002). Most importantly, understanding of the long-term costs and benefits of interventions in chronic illness is key to clinical management and requires knowledge of outcomes over an extended observation period. However, the longer the observation period, the less likely SEP assessment is representative of a patient's clinical state.

This study describes the performance of alternative metrics for quantifying antidepressant effects in terms of their strength of association with change in QoL in a large sample of patients with DTD. The sample consisted of participants in a long-term (5-year), prospective, observational, multi-center outcome registry (D-23 registry) who received treatment-as-usual (TAU) and who were treated with or without adjunctive vagus nerve stimulation (VNS). Individuals were identified who participated in the registry for at least one year and clinical outcomes over the first 2 years of follow-up were examined. All metrics were computed for scores on both the clinician-rated Montgomery Åsberg Depression Rating Scale (MADRS) (Montgomery & Åsberg, 1979) and the patient-rated Quick Inventory of Depressive Symptoms-Self Report (QIDS-SR) (Rush et al., 2003c; Rush, Carmody, & Reimitz, 2006a).

We computed traditional SEP metrics corresponding to the raw symptom severity score at exit or completion of the 2-year observation period, the percentage change from baseline in this score, and binary classifications of partial response, response, and remission. We contrasted these ‘SEP metrics’ with ‘integrative (INT) metrics’ that averaged or aggregated scores over the entire observation period. The INT metrics, in turn, were either severity-based or time-based. The severity-based INT metrics were the median severity score during the 2-year observation period and the median percentage change in these scores relative to baseline. The time-based INT metrics were the percentage of months during the observation period that the patient met criteria for partial response, response, or remission.

We first contrasted the performance of these metrics in separating participants who did or did not achieve a threshold level of meaningful QoL improvement at the end of the observation period – a Minimal Important Clinical Difference (MICD) (Conway et al., 2018; Endicott, Rajagopalan, Minkwitz, Macfadden, & Group, 2007; Jaeschke, Singer, & Guyatt, 1989). We computed the effect size (ES) for the comparison of the binary QoL outcome groups in scores on each metric (*t* tests or tests of proportions). Similarly, we contrasted the metrics in their capacity to discriminate between the QoL groups by computing signal detection parameters [area under the curve (AUC), sensitivity, specificity] derived from receiver operating characteristic (ROC) analyses. We expected that the remission classification, having the highest threshold for demarking improvement, would have the weakest performance characteristics (smallest ES and AUC), while the partial response classifications would have the strongest performance characteristics in detecting QoL categorical improvement. We also expected that the INT metrics would provide more reliable and valid indicators of persistent improvement and would have stronger performance characteristics than the comparable SEP metrics in predicting improved QoL. To confirm the findings based on the binary categorization of QoL outcome groups, we also conducted simultaneous regression analyses predicting the final continuous Q-LES-Q-SF score, based on each metric, with and without adjusting for relevant covariates.

This study made minimal assumptions when formulating outcome metrics and did not attempt to optimize any metric using imputation, weighting, or other techniques. This study provides descriptive information about the strength of the relationship of each metric to QoL improvement and addresses the questions of whether lower thresholds for symptomatic improvement and information integrated over time have stronger relations to QoL outcomes than traditional binary outcome classifications and SEP assessment.

## Methods

### Sample

The D23 registry (ClinicalTrials.gov Identifier: NCT00320372) participants were 18 years and older and in a current MDE by Mini International Neuropsychiatric Interview (Sheehan et al., 1998) and DSM-IV-TR (First & Pincus, 2002) criteria. The current MDE (unipolar or bipolar depression) was at least 2 years in duration or the participant had a history of least three MDEs, including the current episode. Participants also demonstrated lack of response to four or more adequately delivered antidepressant treatments, defined as the minimal therapeutic dose per the Food and Drug Administration (FDA) Physicians' Desk Reference (PDR) labeling for a minimum of 4 weeks, or non-response to a course of electroconvulsive therapy (ECT) or evidence-based psychotherapy. Inclusion also required a baseline Clinical Global Impression Severity (CGI-S) (Guy, 1976) score of at least 4 and no history of schizophrenia, schizoaffective disorder or other psychotic disorder, rapid cycling bipolar disorder, previous use of VNS therapy, or current psychotic features.

The registry included 61 sites in the USA representing a mix of academic, institutional, and private clinic settings. Registry participation was approved by an institutional review board. Written informed consent was obtained from all participants. Registry data were collected between January 2006 and May 2015. Details regarding the treatment and assessment of registry participants are provided elsewhere (Aaronson et al., 2017). The intent was to follow the natural course of DTD in the TAU group, and any psychopharmacologic, neurostimulation, or psychotherapeutic intervention could be administered over the 5-year study period to any patient.

The D-23 registry enrolled an intent-to-treat (ITT) sample of 606 participants (*n* = 330 VNS + TAU; *n* = 276 TAU). In some reports on long-term outcomes, a separate group of 159 patients who completed a D-21 protocol were added to the D-23 registry sample (Aaronson et al., 2017). As this ‘rollover’ group did not have the necessary QoL assessment at baseline, they were not included in this study. We excluded from this report participants who had either a missing baseline assessment or dropped out of the study before the 12-month follow-up (*n* = 5). We also excluded participants with a baseline MADRS score < 18 (*n* = 12), baseline QIDS-SR score < 10 (*n* = 30), or both (*n* = 12) to ensure at least moderate baseline symptom severity on each measure. We also excluded participants (*n* = 141) who did not complete the MADRS, QIDS-SR, and the Quality of Life, Enjoyment and Satisfaction Questionnaire – Short Form (Q-LES-Q-SF) (Endicott, Nee, Harrison, & Blumenthal, 1993) on at least one occasion on or after the 12-month follow-up visit. These restrictions resulted in a final sample of 406 participants (*n* = 234 VNS + TAU; *n* = 172 TAU). A CONSORT flowchart of participant disposition is provided in online Supplementary Fig. S1.

### Depression severity and quality-of-life measures

The MADRS is a 10-item clinician-rated scale that assesses the severity of depressive symptoms in patients with mood disorders (Montgomery & Åsberg, 1979). Each item was rated on a scale of 0–6 for symptom manifestation over the past week. Suggested severity ranges for total scores are: 0–6 no depression; 7–19 mild depression; 20–34 moderate depression, and 34–60 severe depression (Carmody et al., 2006). The QIDS-SR is a 16-item, self-report instrument that also assesses depressive symptom severity over the past week (Rush et al., 2003b; Rush et al., 2006a). Items were rated on a scale of 0–3. Suggested severity ranges for total scores are 0–5 no depression; 6–10 mild depression; 11–15 moderate depression; 16–20 severe depression; and 21–27 very severe depression (Cameron et al., 2013). The Q-LES-Q-SF is a self-report scale that assesses the degree of enjoyment and satisfaction experienced by the participant during the past week. The short form documents satisfaction in 14 content domains (e.g. household activities; family relationships), followed by two global ratings (e.g. life satisfaction and contentment). The items are rated on a scale of 1–5 and the scores on the 14 content domains summed, producing raw total scores ranging from 14 (worst) to 70 (best) (Endicott et al., 1993; Endicott et al., 2007). The Q-LES-Q-SF has excellent internal consistency and test-retest reliability (Rapaport, Clary, Fayyad, & Endicott, 2005; Wyrwich et al., 2009). While its content applies to QoL in the general population, it has shown sensitivity to clinical presentation and treatment outcomes in generalized anxiety disorder (Demyttenaere, Andersen, & Reines, 2008; Wyrwich et al., 2009), bipolar disorder (Calabrese et al., 2016; Wyrwich et al., 2011), and major depressive disorder (Demyttenaere et al., 2008), including DTD (Conway et al., 2018).

### Assessments, metrics, and quality-of-life outcome groups

Assessments were conducted at baseline (visit prior to surgical implantation of VNS), at 3, 6, 9, 12, 18, and 24 months post-baseline, and every 6 months thereafter until study exit at 60 months. The observation period used in this study was limited to the first 24 months post-baseline, since there were considerable missing data after 24 months, and starting at 12 months post-baseline, assessments were conducted at only 6-month intervals. The QIDS-SR and Q-LES-Q were completed on-site at these visits. After each on-site visit, the site notified central raters to initiate a patient telephone follow-up. The central raters were trained clinicians who conducted the MADRS assessments (Aaronson et al., 2017).

**Fig. 1.**
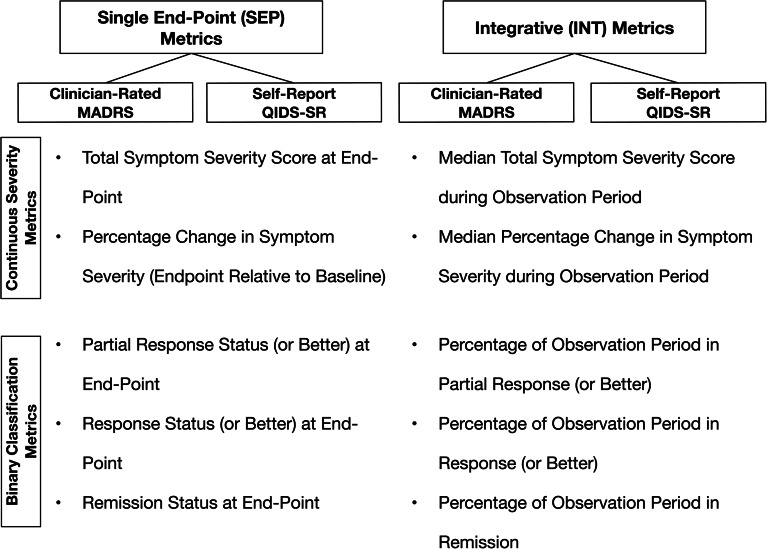
Alternative outcome metrics grouped by whether clinical outcome was assessed at a single end-point (SEP) or by integrating symptom scores over an observation period (INT). Metrics were also grouped as either continuous measures of symptom severity or binary classifications of the extent of improvement. Each metric was computed for both a clinician-rated and a self-report depression symptom severity scale.

SEP outcome metrics were based on the last observed MADRS or QIDS-SR total score. These metrics, computed for both instruments, included (1) the total symptom severity score at the end of the observation period; (2) the percentage change in this score relative to baseline [(pre-post)/pre] × 100; (3) partial response status, defined as a percentage change ⩾35%; (4) response status, defined as a percentage change ⩾50%; and (5) remission status, defined as an endpoint score ⩽9 for the MADRS and ⩽5 for the QIDS-SR (Figure 1).

The INT metrics incorporated all available scores during the observation period (post-baseline 3, 6, 9, 12, 18, 24-month visits). These metrics were divided into severity-based and time-based measures, and each INT metric corresponded to a specific SEP metric, but now averaging (severity-based) or aggregating (time-based) scores over time (Figure 1). The INT severity-based metrics were (1) the median of total scores over the post-baseline observation period and (2) the median percentage change from baseline in total scores as computed at each post-baseline visit. The INT time-based metrics were the proportion of the observation period that the participant met the threshold for (3) partial response, (4) response, and (5) remission. In calculating these proportions, the number of months was determined that intervened between the first assessment at which a participant met the criterion for an outcome category (e.g. remission) and the first subsequent assessment when the criterion was not met (e.g. non-remission), missing visit, or study discontinuation, whichever occurred first. Thus, no interpolation was used for missing information in calculating these proportions.

These metrics were compared in the strength of their relationships with change in QoL. Endicott et al. (2007) suggested that for the Q-LES-Q-SF an increase of 11.89% or more relative to baseline (in the percentage maximum score) corresponded to a MICD, distinguishing those with unimproved QoL from those with a minimally clinically meaningful improvement or better. In a treatment trial with a large bipolar depression sample, this value was associated with an end-point Clinical Global Improvement (CGI-I) (Guy, 1976) rating of at least ‘minimally improved’ (scores of 1–3). In the D-23 registry sample examined in this study, Conway et al. (2018) reported that participants in the VNS + TAU group were more likely to meet this MICD threshold than participants in the TAU group. Participants in the VNS + TAU group reliably met this threshold with a MADRS percentage improvement score of at least 36%, considerably below the 50% symptom reduction threshold traditionally used to define clinical response. The raw scores on the Q-LES-Q-SF, ranging from 14 to 70, were rescaled to range from 0 to 100 by computing the percentage maximum score. A threshold improvement in these percentage maximum scores relative to baseline (post–pre) of at least 11.89% defined assignment to the improved and unimproved QoL outcome groups.

### Statistical analyses

The Shapiro–Wilk test was used to screen for departures from normality in continuous demographic and clinical measures and metric scores. The distributions of number of previous MDEs, hospitalizations in the last 5 years, and lifetime suicide attempts were skewed due to high outlying values in some participants. These variables each were capped to a maximal score of 10. The QoL outcome groups (improved *v.* unimproved) were compared in demographic and clinical characteristics using the Wilcoxon rank-sum test for continuous measures and Fisher's exact test for categorical variables.

ES was calculated for each metric, reflecting the magnitude of the standardized difference between the QoL outcome groups in mean metric scores. For all metrics other than the SEP binary classifications (partial response, response, and remission), ES was calculated as the difference between the means of the two groups relative to a pooled standard deviation, Cohen's 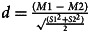. Since participants could only have scores of 0 or 1 for the SEP binary classifications, following Cohen's recommendations (Cohen, 1988), ES was calculated for the difference between the proportions of the two QOL outcome groups, Cohen's 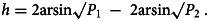 The 95% confidence interval is also reported for each ES.

ES provided a standardized measure to contrast the metrics in the magnitude of the difference between the two QoL outcome groups in mean scores. A related question concerns the extent to which metrics were useful in identifying participants classified as improved or unimproved in QoL, i.e. their accuracy when making this discrimination. ROC curves were generated for each metric in detecting the QoL binary outcome and standard signal detection methods applied to quantify overall performance (AUC), sensitivity (accuracy in detecting QoL improvement), and specificity (accuracy in detecting lack of QoL improvement) (Green & Swets, 1966; McNicol, 2004; Stanislaw & Todorov, 1999). AUC provides an index of the overall performance in distinguishing the groups, where values of 0.5 indicate chance performance and values of 1.0 correspond to errorless detection (Hajian-Tilaki, 2013). From the ROC curve evaluation (Obuchowski, 2005), the best cutoff value of each metric was identified as the point with the highest combined sensitivity and specificity (Youden index) (Fluss, Faraggi, & Reiser, 2005). At this optimal cutoff, the metric's accuracy, sensitivity, and specificity are reported with the corresponding 95% CI derived from the normal approximation.

Wilcoxon non-parametric matched-paired signed rank tests were used to test the consistency of the differences among specific metric groupings. ES and AUC values were compared for the 10 metrics based on the MADRS compared to the 10 respective QIDS-SR metrics. Similarly, the 10 metrics based on SEP assessment were compared to their respective INT metrics.

The differences among the metrics in the foregoing analyses could be specific to the threshold used to define MICD QoL binary outcome groups. Another set of analyses described the strength of association between each metric and the continuous end-point Q-LES-Q-SF total scores. A set of ‘simple’ simultaneous regression analyses was performed on these scores with the baseline Q-LES-Q-SF score and the individual metric as independent variables (Cohen, Cohen, West, & Aiken, 2003). A parallel set of ‘expanded’ regression analyses added treatment condition (VNS + TAU *v.* TAU), age, gender, duration of current episode, number of lifetime depressive episodes, and number of psychiatric hospitalizations in the past 5 years as additional covariates. The strength of each metric's association with the continuous QoL outcome was assessed with the standardized regression coefficient (*β*) which quantified the strength of the relationship between the metric and the Q-LES-Q-SF scores after each variable has been standardized. This coefficient is ‘unitless’ and allows comparison across metrics with different scaling (Newman & Browner, 1991).

The analyses of ES, discriminability, and the prediction of continuous end-point QoL scores indicated that a specific SEP severity-based metric and a specific INT time-based metric had strong associations with binary and continuous QoL outcomes. To determine whether each metric contributed unique variance, the simultaneous multiple regressions analyses described above were repeated including both metrics as independent variables.

## Results

### Sample characteristics

Across the sample, the prototypic patient presented with a severe and chronic MDE, and a history of multiple prior MDEs, psychiatric hospitalizations, and suicide attempts (Table 1). The sample averaged approximately eight adequate but ineffective treatment trials for the MDE and more than 50% of participants had received ECT in the past. As seen in Table 1, 153 of the 406 (37.7%) participants were classified as meeting the MICD threshold for improvement in Q-LES-Q-SF scores at end-point. The QoL outcome groups differed significantly only in baseline Q-LES-Q-SF scores. The baseline score was lower in those who were classified as improved.

**Table 1.** Demographics and clinical characteristics of the total sample and the improved and unimproved quality-of-life (QoL) outcome groups

	Total sample *N* = 406		QoL improved *N* = 153		QoL unimproved *N* = 253		*p*
	Mean	s.d.	Mean	s.d.	Mean	s.d.	
Age (yr)	49.57	10.22	50.08	9.41	49.26	49.57	0.60
Baseline MADRS	32.31	6.64	33.03	6.99	31.87	6.39	0.09
Baseline QIDS-SR	18.10	4.09	18.54	4.19	17.83	4.01	0.10
Baseline Q-LES-Q-SF (% maximum)	15.11	4.60	13.38	3.96	16.15	4.66	<0.0001
Duration current episode (yr)	8.26	10.03	7.39	9.24	8.78	10.47	0.10
Age at first depression diagnosis (yr)	29.13	10.96	29.37	11.61	28.99	10.57	0.84
No. lifetime MDE^a^	5.30	3.63	5.58	3.63	5.13	3.63	0.19
No. psychiatric hospitalizations in past 5 years^a^	1.99	2.66	2.16	2.62	1.89	2.68	0.10
No. lifetime suicide attempts^a^	1.44	2.34	1.63	2.53	1.32	2.21	0.28
No. lifetime failed courses of MDE treatment	7.98	3.11	7.85	2.89	8.05	3.24	0.77
	*N*	%	*N*	%	*N*	%	
Gender (*N*, % female)	269	66.3%	103	67.3%	166	65.6%	0.75
Treatment group (*N*, % VNS + TAU)	234	57.6%	100	65.4%	134	53.0%	0.11
Bipolar MDE (*N*, % bipolar)	97	23.9%	43	28.1%	54	21.3%	0.15
Age at depression diagnosis ⩽18 yr (*N*, %)	72	17.7%	29	19.0%	43	17.0%	0.69
Received ECT lifetime (*N*, % ECT)	223	55.1%	90	59.2%	133	52.6%	0.22

a Maximum score of 10 applied. *p* values refer to the significance level of the contrast of the QoL improved and unimproved groups using the Wilcoxon rank-sum test for continuous measures and Fisher's exact test for categorical variables.

Participants averaged more than 22 months of follow-up (out of a maximum of 24 months) and the MADRS and QIDS-SR were completed at more than five out of six possible follow-up visits (online Supplementary Table S1). There was no difference between the QoL outcome groups in length of follow-up or number of symptom severity assessments.

### Metric performance: effect size and discriminability of QoL outcome groups

**Fig. 2.**
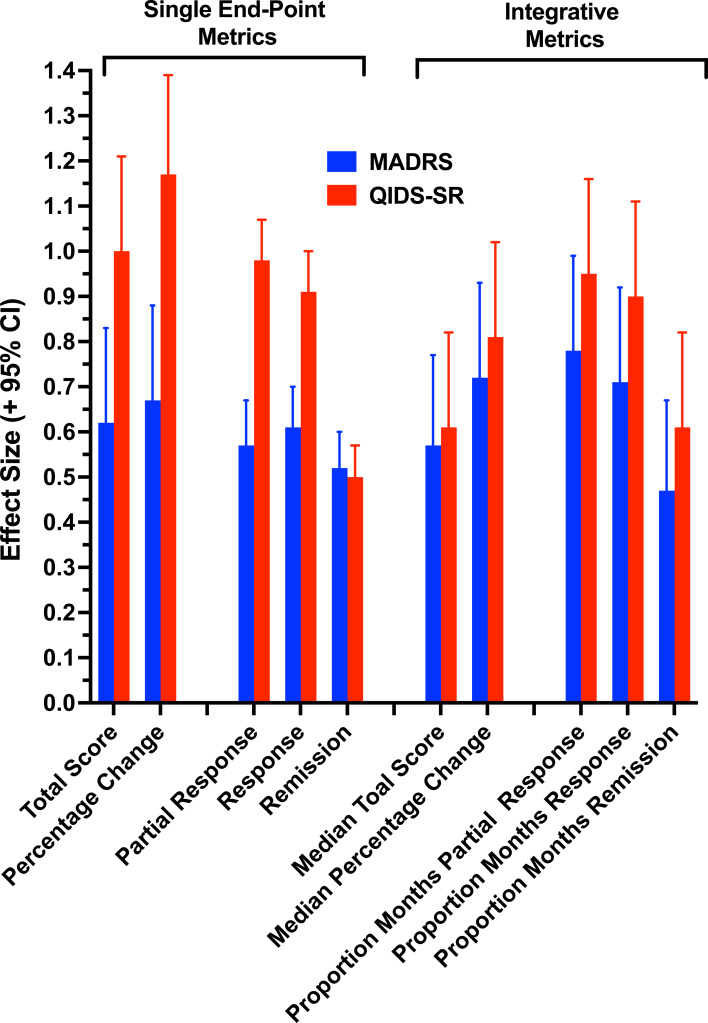
Effect size with 95% confidence interval for the comparison of the improved and unimproved quality-of-life groups in symptom improvement on each single end-point and integrative outcome metric.

Table 2 presents the descriptive statistics for the binary QoL outcome groups on each metric, as well as the ES of the difference between the means (Cohen's *d*) or proportions (Cohen's *h*). Figure 2 displays the ES for all metrics in distinguishing the QoL outcome groups. Table 3 presents the signal detection parameters derived from the discrimination of the binary QoL outcomes based on each metric. Since the results were consistent across the ES and AUC measures, the findings are discussed together.

**Table 2.** Effect sizes of the metrics in separating the improved (achieved MICD) and unimproved (did not achieve MICD) quality-of-life (QoL) outcome groups

	Total sample *N* = 406		QoL improved *N* = 153		QoL unimproved *N* = 253		Effect size Cohen's *d*/*h*	Effect size 95% CI
	Mean	s.d.	Mean	s.d.	Mean	s.d.		
Single end-point metrics								
Total MADRS score^a^	22.18	10.44	18.33	10.55	24.51	9.67	0.62	0.41–0.83
Percentage change in MADRS score^a^	29.90	33.06	43.02	33.55	21.97	30.16	0.67	0.46–0.88
Total QIDS-SR score^a^	13.24	5.83	9.97	5.32	15.23	5.21	1.00	0.79– 1.21
Percentage change in QIDS-SR score^a^	25.52	32.46	46.09	26.41	13.08	29.33	1.17	0.95–1.39
Single end-point binary outcome metrics								
Partial response MADRS (*N*, %)^b^	163	40.1%	88	57.5%	75	29.6%	0.57	0.47–0.67
Response MADRS (*N*, %)^b^	113	27.8%	69	45.1%	44	17.4%	0.61	0.52–0.70
Remission MADRS (*N*, %)^b^	55	13.5%	38	24.8%	17	6.7%	0.52	0.44–0.60
Partial response QIDS-SR (*N*, %)^b^	158	38.9%	104	68.0%	54	21.3%	1.07	0.89–1.07
Response QIDS-SR (*N*, %)^b^	108	26.6%	79	51.6%	29	11.5%	0.91	0.82–1.00
Remission QIDS-SR (*N*, %)^b^	39	9.6%	29	19.0%	10	4.0%	0.50	0.43–0.57
Integrative severity-based metrics								
Median of MADRS total scores^a^	23.51	8.58	20.56	9.12	25.30	7.73	0.57	0.37–0.77
Median percentage change in MADRS scores^a^	26.04	25.41	36.81	26.33	19.54	22.50	0.72	0.51–0.93
Median of total QIDS-SR scores^a^	13.99	4.95	12.19	4.92	15.09	4.65	0.61	0.40–0.82
Median percentage change in QIDS-SR scores^a^	21.27	26.60	33.77	23.29	13.71	25.65	0.81	0.60–1.02
Integrative time-based binary outcome metrics								
Proportion of months in MADRS partial response^a^	0.37	0.39	0.55	0.38	0.27	0.35	0.78	0.57–0.99
Proportion of months in MADRS response^a^	0.25	0.35	0.40	0.39	0.16	0.30	0.71	0.50–0.92
Proportion of months in MADRS remission^a^	0.10	0.23	0.17	0.29	0.06	0.18	0.47	0.27–0.67
Proportion of months in QIDS-SR partial response^a^	0.35	0.36	0.55	0.37	0.23	0.30	0.95	0.74–1.16
Proportion of months in QIDS-SR response^a^	0.23	0.32	0.39	0.37	0.13	0.24	0.90	0.69–1.11
Proportion of months in QIDS-SR remission^a^	0.09	0.21	0.16	0.27	0.04	0.13	0.61	0.40–0.82

MADRS, Montgomery Åsberg Depression Rating Scale; QIDS-SR, Quick Inventory of Depressive Symptoms-Self Report; MICD, Minimally Important Clinical Difference.

a Effect size was calculated as Cohen's *d.*

b Effect size was calculated as Cohen's *h.*

**Table 3.** Signal detection parameters for the discrimination of participants with (*N* = 153) and without (*N* = 252) MICD improved quality-of-life (QoL) on the basis of metric scores

Metric	AUC	95% CI	Sensitivity	Specificity
Single end-point severity-based metrics				
Total MADRS score	0.67	0.62–0.73	0.58	0.72
Percentage change in MADRS score	0.69	0.63–0.74	0.55	0.78
Total QIDS-SR scores	0.76	0.70–0.83	0.73	0.72
Percentage change in QIDS-SR score	0.81	0.77–0.85	0.80	0.72
Single end-point binary outcome metrics				
Partial response MADRS	0.64	0.59–0.69	0.58	0.70
Response MADRS	0.64	0.59–0.68	0.45	0.83
Remission MADRS	0.59	0.55–0.63	0.25	0.93
Partial response QIDS-SR	0.73	0.69–0.78	0.68	0.79
Response QIDS-SR	0.70	0.66–0.75	0.52	0.89
Remission QIDS-SR	0.58	0.54–0.61	0.19	0.96
Integrative severity-based metrics				
Median of MADRS total scores	0.66	0.61–0.72	0.64	0.64
Median percentage change in MADRS scores	0.70	0.64–0.75	0.67	0.66
Median total QIDS-SR scores	0.67	0.61–0.72	0.62	0.67
Median percentage change in QIDS-SR scores	0.72	0.67–0.77	0.80	0.56
Integrative time-based binary outcome metrics				
Proportion of months in MADRS partial response	0.70	0.65–0.76	0.77	0.58
Proportion of months in MADRS response	0.67	0.62–0.73	0.55	0.79
Proportion of months in MADRS remission	0.61	0.56–0.65	0.35	0.86
Proportion of months in QIDS-SR partial response	0.74	0.69–0.79	0.75	0.65
Proportion of months in QIDS-SR response	0.71	0.66–0.76	0.60	0.78
Proportion of months in QIDS-SR remission	0.62	0.57–0.66	0.34	0.90

AUC, area under the receiver operating characteristic (ROC) curve; MADRS, Montgomery Åsberg Depression Rating Scale; MICD, Minimally Important Clinical Difference; QIDS-SR, Quick Inventory of Depressive Symptoms-Self Report.

ES and AUC were substantially greater for metrics based on the QIDS-SR compared to comparable MADRS metrics. The paired comparisons of MADRS and QIDS-SR metrics yielded significant differences in ES (*p* < 0.004) and AUC (*p* < 0.008), indicating greater separation of QOL outcome groups with QIDS-SR relative to MADRS metrics. Across SEP and INT metrics, percentage change in total scores had higher ES and AUC values than the raw total scores. Indeed, the SEP metric of percentage change in QIDS-SR scores had the highest ES (1.17) and AUC (0.81) values across all SEP and INT metrics.

We expected that most restrictive clinical outcome classification (i.e. remission) would have poorer performance characteristics than classifications using more liberal thresholds (e.g. partial response). This expectation was supported across SEP and INT metrics, whether examining MADRS or QIDS-SR scores. ES and AUC values were notably lower for metrics based on remission than metrics based on response or partial response classifications. Sensitivity in detecting improved QoL was lowest, though specificity highest, for remission compared with partial response and response classifications. Indeed, it was evident from the signal detection analyses (Table 3) that sensitivity dropped more precipitously than specificity increased when comparing partial response, response, and remission metrics, accounting for the overall poorer performance of the remission classification. Across the SEP and INT metrics, relative to response and remission, the partial response classification had the strongest performance indices in separating and classifying the binary QoL outcome groups.

Contrary to our expectation, as overall groupings, the SEP and INT metrics did not differ in ES (*p* = 0.63) or AUC (*p* = 0.58) values. Indeed, the SEP severity-based measures showed stronger associations with QoL outcome group than the corresponding INT severity-based metrics. Symptom severity scores at final evaluation had stronger relationships with the QoL outcome classification than the median of symptom severity scores over the observation period. In contrast, the INT time-based metrics, quantifying the proportion of the observation period meeting threshold levels of symptom improvement, generally had stronger performance characteristics than the respective SEP outcome classifications. Similarly, within the SEP metrics, ES and AUC were superior for the severity-based measures compared to the binary outcome classifications. However, within the INT metrics, the pattern was reversed, and the time-based metrics based on aggregated binary outcome classification had considerably stronger performance characteristics than the INT severity-based measures. Within the INT time-based metrics, the proportion of months in QIDS-SR partial response had the highest ES (0.95) and AUC (0.74) values.

### Metric performance: association with continuous QoL outcome

Simultaneous linear regression analyses were conducted on continuous end-point Q-LES-Q total scores. In the ‘simple’ analyses only baseline Q-LES-Q scores and scores for the individual metrics were predictors. In ‘expanded’ analyses, six demographic and clinical variables were added as additional predictors in each model. The findings for the simple and expanded analyses were indistinguishable, with virtually identical standardized regression coefficients for each metric and the increase in the total variance accounted for in the expanded models ranging from only 1% to 3%. Table 4 presents the results of the simple analyses.

**Table 4.** Multiple linear regression analyses on continuous end-point Q-LES-Q-SF scores with each metric and baseline Q-LES-Q-SF scores as predictors

	*b*	95% CI	*ß*	*p*	Total model *R*^2^
Single end-point severity-based metrics					
Total MADRS score	−0.28	−0.33 to −0.23	−0.49	<0.0001	0.31
Percentage change in MADRS score	0.07	0.06–0.09	0.41	<0.0001	0.26
Total QIDS-SR scores	−0.72	−0.79 to −0.65	−0.70	<0.0001	0.56
Percentage change in QIDS-SR score	0.12	0.10–0.13	0.63	<0.0001	0.49
Single end-point binary outcome metrics					
Partial response MADRS	4.40	3.34–5.46	0.36	<0.0001	0.22
Response MADRS	5.06	3.90–6.21	0.38	<0.0001	0.23
Remission MADRS	6.10	4.57–7.62	0.35	<0.0001	0.21
Partial response QIDS-SR	6.95	6.02–7.87	0.57	<0.0001	0.41
Response QIDS-SR	7.60	6.57–8.62	0.56	<0.0001	0.41
Remission QIDS-SR	6.82	5.02–8.61	0.34	<0.0001	0.20
Integrative severity-based metrics					
Median of MADRS total scores	−0.34	−0.40 to −0.28	−0.49	<0.0001	0.31
Median percentage change in MADRS scores	0.10	0.08–0.12	0.41	<0.0001	0.26
Median total QIDS-SR scores	−0.71	−0.81 to −0.61	−0.58	<0.0001	0.39
Median percentage change in QIDS-SR scores	0.11	0.09–0.13	0.48	<0.0001	0.32
Integrative time-based binary outcome metrics					
Proportion of months in MADRS partial response	6.63	5.34–7.93	0.43	<0.0001	0.27
Proportion of months in MADRS response	7.28	5.86–8.71	0.43	<0.0001	0.27
Proportion of months in MADRS remission	9.10	6.82–11.37	0.35	<0.0001	0.21
Proportion of months in QIDS-SR partial response	9.00	7.73–10.27	0.54	<0.0001	0.39
Proportion of months in QIDS-SR response	9.86	8.39–11.33	0.53	<0.0001	0.37
Proportion of months in QIDS-SR remission	10.71	8.17–13.25	0.37	<0.0001	0.23

*b*, unstandardized regression coefficient with 95% confidence interval; *ß*, standardized regression coefficient; total model *R*^2^ (overall goodness-of-fit, coefficient of determination) = amount of variance in Q-LES-Q scores accounted for by the total model (metric and baseline Q-LES-Q-SF).

As in the preceding analyses on ES and AUC in separating binary QoL outcome groups, performance characteristics (standardized regression coefficients, *ß*) were superior for QIDS-SR compared to corresponding MADRS metrics. Within the SEP and INT metrics based on binary outcome classification, the weakest associations with continuous end-point QoL scores were found for the remission metrics compared to partial response and response metrics, which were generally equivalent. This pattern was especially marked for QIDS-SR scores. As before, INT severity metrics had weaker associations than SEP severity metrics using the QIDS-SR, but equivalent values using the MADRS. INT time-based metrics had stronger associations than SEP outcome classifications based on the MADRS and equivalent values based on the QIDS-SR.

Table 5 presents the results of the regression analyses predicting the continuous end-point QoL score on the basis of the baseline score, percentage change in symptom severity, and proportion of months in partial response. For both the MADRS and QIDS-SR, each metric significantly contributed unique variance. For MADRS scores, the strength of association was stronger for the INT time-based metric than the SEP severity measure, while the opposite was the case for QIDS-SR scores.

**Table 5.** Multiple linear regression analyses on continuous end-point Q-LES-Q-SF scores with baseline Q-LES-Q-SF scores, and optimal single send-point (SEP) and integrative (INT) metrics as predictors

	*b*	95% CI	*ß*	*p*	Total model *R*^2^
MADRS					0.29
Baseline Q-LES-Q-SF score	0.33	0.23–0.44	0.26	<0.0001	
Percentage change in total score	0.03	0.01–0.06	0.19	0.002	
Proportion of months in partial response	4.49	2.63–6.35	0.29	<0.0001	
QIDS-SR					0.52
Baseline Q-LES-Q-SF score	0.42	0.33–0.51	0.32	<0.0001	
Percentage change in total score	0.09	0.07–0.11	0.49	<0.0001	
Proportion of months in partial response	3.26	1.63–4.89	0.20	0.0001	

*b*, unstandardized regression coefficient with 95% confidence interval; *ß*, standardized regression coefficient; total model *R*^2^ (overall goodness-of-fit, coefficient of determination) = amount of variance in Q-LES-Q scores accounted for by the total model.

## Discussion

It is often stated that symptomatic remission is the goal of acute antidepressant treatment (Gelenberg, 2010; Möller, 2008; Rush et al., 2006b). Multiple investigations have used remission rate as their primary efficacy outcome, including studies in TRD (Dean et al., 2021; O'Reardon et al., 2007; Rush et al., 2006c; Sackeim et al., 2009). However, in line with our expectations, this study found that, relative to the binary classifications of partial response and response, SEP and INT metrics based on remission had the smallest ESs in distinguishing those who did or did not meet our *a priori* defined threshold of improvement in QoL. Indeed, remission-based metrics also had the lowest classification accuracy in the signal detection analyses, and the weakest associations with continuous QoL scores at end-point in regression analyses. Thus, in clinical treatment studies of DTD, where achieving remission is infrequent or transient, if obtained, reliance on this binary (remission/non-remission) classification may also be insensitive in identifying clinically useful interventions, as originally suggested by Rush et al. (2003a). It is noteworthy that in psychiatric disorders with a chronic course and infrequent periods of symptomatic remission, such as obsessive–compulsive disorder and schizophrenia, symptom improvement thresholds between 25% and 35% are often used to ascribe positive clinical outcome in primary efficacy analyses (Bighelli et al., 2018; Burchi, Hollander, & Pallanti, 2018; Leucht, Davis, Engel, Kissling, & Kane, 2009; Mataix-Cols et al., 2016).

We also expected that metrics that integrated information over time would have stronger performance characteristics than those based on an SEP. This idea was not supported when comparing the SEP and INT severity metrics. Especially with the QIDS-SR, the SEP severity metrics had stronger associations with QoL outcomes than the INT severity-based measures. That is, symptom severity at the time of QoL assessment had a stronger association with QoL scores than the median of symptom severity scores over the observation period. In contrast, the INT time-based metrics generally had equivalent or stronger relations with QoL outcomes than the comparable SEP binary classifications. This is likely a result of the INT time-based metrics aggregating information about symptom improvement over time, thereby incorporating a component reflecting durability of benefit, in contrast to the INT severity-based measures, which essentially took an ‘average snapshot’ of symptom severity. The final set of regression analyses demonstrated that, when considered together, an optimal SEP severity metric and an optimal INT time-based metric each significantly contributed unique variance in accounting for end-point QoL scores.

These findings suggest reconsideration of the outcome metrics used in primary and secondary analyses of intervention trials in DTD. It is widely accepted that the fundamental goal of medical treatment is to produce sustained improvement in patients' QoL. Symptom improvement is but a means toward this end, as is the minimization of side effect burden (Megari, 2013; Wilson & Cleary, 1995). It was evident in this study that relative to lower thresholds of symptom improvement, the remission classification was insensitive in detecting a large proportion of individuals with and without meaningful QoL improvement, as well as predicting the magnitude of QoL change. Integrating information about partial response or response over time also generally had stronger relations to QoL outcomes than the comparable SEP binary classifications. Thus, in addition to traditional SEP symptom severity measures, INT time-based metrics based on partial response or response should be considered as outcome metrics when designing intervention trials in DTD and perhaps other chronic, difficult-to-treat conditions (Conway et al., 2020). Of note, this study examined strength of association when the metrics were used as independent variables detecting and predicting QoL outcomes. In a subsequent study, we will compare the alternative metrics in their utility in revealing intervention effects, contrasting the VNS + TAU group with the TAU only group in SEP and INT metric scores.

This study has important limitations. The metric comparisons were principally descriptive and reflected patterns in a single patient sample. Observations, such as the diminished sensitivity to QoL outcomes when using the remission classification, could not be tested for statistical significance and multiple studies of this type, using meta-analytic techniques to contrast metrics, will be needed to determine whether the patterns obtained here generally apply in DTD. Nonetheless, the magnitude of the differences observed among the metrics, and particularly the poor performance of metrics based on remission, underscores the need for re-thinking outcome criteria in this subgroup. The performance of the metrics was tested only against QoL binary and continuous outcomes determined at an SEP. It is possible that the performance of the INT metrics would be further enhanced had an INT QoL outcome measure been used. The metrics based on the QIDS-SR consistently had stronger relations with the QoL outcomes than the comparable metrics based on the MADRS. The shared method variance due to the use of self-report for both the QIDS-SR and the QoL measures may account for this specification (Podsakoff, MacKenzie, Lee, & Podsakoff, 2003; Spector, Rosen, Richardson, Williams, & Johnson, 2019). Traditionally ESs for efficacy outcomes in depression treatment trials are often smaller for self-report than clinician-rated scales (Lin, Lu, Wong, & Chen, 2014; Prusoff, Klerman, & Paykel, 1972; Sayer et al., 1993). Comparison of the metrics in their sensitivity to treatment effects is needed. The relatively stringent cutoffs used to define remission on the symptom scales may have contributed to the poor performance of remission-based metrics (Hawley, Gale, & Sivakumaran, 2002; Zimmerman, Posternak, & Chelminski, 2004). The symptom severity measures were only obtained at 3–6 months intervals. Use of more frequent self-report assessment should be investigated as a means to optimize INT time-based metrics. Finally, this study compared metric performance when characterizing longer-term outcomes and required a follow-up period of 12–24 months. Approximately 25% of the entry sample was excluded since they did not have a complete set of MADRS, QIDS-SR, and Q-LES-Q ratings within this time period. The comparative performance of the metrics was not tested with shorter follow-up intervals, as would occur in an ITT sample.

The DTD sample was amongst the most chronic and treatment-resistant ever studied. The findings demonstrated that integrating information about symptom severity and using lower thresholds for binary classifications of symptom improvement enhance sensitivity in detecting improved QoL, the overall goal of treatment interventions. It is unknown whether the same relations hold in less chronic and treatment-resistant samples.

Surprisingly, across chronic illnesses there has been a paucity of empirical research contrasting alternative metrics for characterizing long-term clinical outcomes in symptom measures (Francis, Dunt, & Cadilhac, 2016; Nolte & Osborne, 2013). The novel methods used in this study to compare outcome metrics in DTD in their strength of association with change in QoL may be applied in other chronic conditions. The findings here prompt greater consideration of INT time-based metrics based on partial response and/or response status to characterize long-term effects on symptom expression. The value of frequently collected patient-reported symptom severity measures deserves further investigation.

## Supplementary material

For supplementary material accompanying this paper visit https://doi.org/10.1017/S0033291722003798. click here to view supplementary material
