# Supplementary material for: Alternative metrics for characterizing longer-term clinical outcomes in difficult-to-treat depression: I. Association with change in quality of life
Source: Psychol Med. 2023 Jan 5;53(14):6511–23. doi: 10.1017/S0033291722003798 (PMC10600942; doi:10.1017/S0033291722003798)
Supplement: Supplementary file 1 [file S0033291722003798sup.zip › S0033291722003798sup001.docx]

Supplemental Table 1. Length of follow-up and number of post-baseline assessments in the total sample and the improved and unimproved Quality-of-Life (QoL) outcome groups.

|  | Total Sample  N = 406 | | QoL Improved  N = 153 | | QoL Unimproved  N = 253 | | *P* |
| --- | --- | --- | --- | --- | --- | --- | --- |
|  | Mean | SD | Mean | SD | Mean | SD |  |
| Q-LES-Q-SF  Final Assessment (mo) | 22.10 | 4.17 | 22.43 | 3.86 | 21.90 | 4.34 | 0.13 |
|  |  |  |  |  |  |  |  |
| MADRS |  |  |  |  |  |  |  |
| Final Assessment (mo) | 22.79 | 4.78 | 22.92 | 4.77 | 22.71 | 4.80 | 0.52 |
| No. post-baseline assessments | 5.22 | 0.98 | 5.19 | 1.01 | 5.24 | 0.96 | 0.74 |
|  |  |  |  |  |  |  |  |
| QIDS-SR |  |  |  |  |  |  |  |
| Final Assessment (mo) | 22.68 | 3.93 | 22.88 | 3.77 | 22.55 | 4.03 | 0.22 |
| No. post-baseline assessments | 5.53 | 0.76 | 5.54 | 0.78 | 5.53 | 0.75 | 0.77 |

P-values refer to the significance level of the contrast of the QoL Improved and Unimproved groups using the Wilcoxon rank-sum test.
