# Supplementary figures and images for: Alternative metrics for characterizing longer-term clinical outcomes in difficult-to-treat depression: I. Association with change in quality of life
Source: Psychol Med. 2023 Jan 5;53(14):6511–23. doi: 10.1017/S0033291722003798 (PMC10600942; doi:10.1017/S0033291722003798)

Supplementary Figure 1. CONSORT Diagram of Participant Disposition

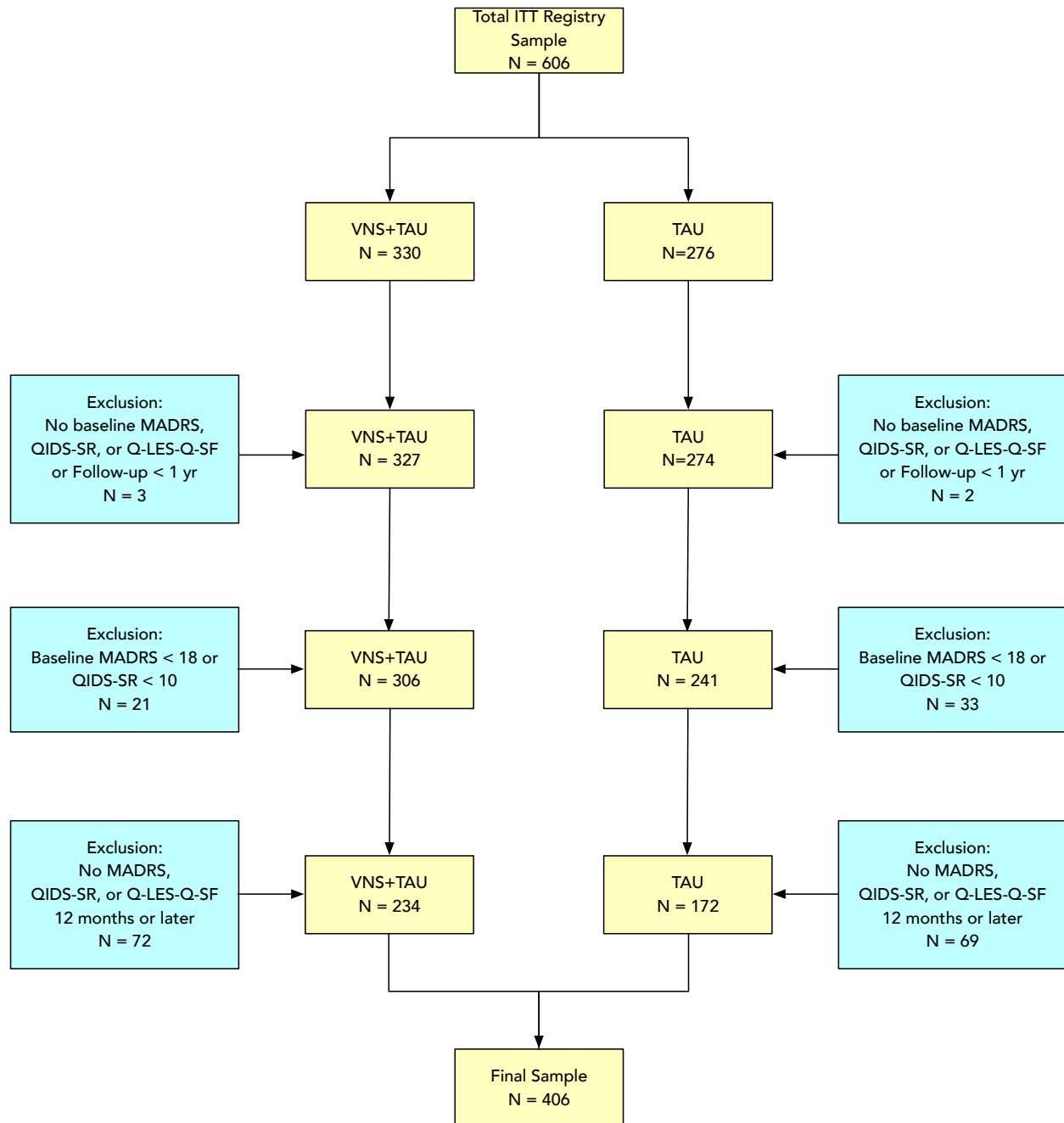

Supplement: Supplementary file 1 [file S0033291722003798sup.zip › S0033291722003798sup002.pdf]
